# Direct Phenotypical and Functional Dysregulation of Primary Human B Cells by Human Immunodeficiency Virus (HIV) Type 1 *In Vitro*

**DOI:** 10.1371/journal.pone.0039472

**Published:** 2012-07-02

**Authors:** Ana Judith Perisé-Barrios, María Ángeles Muñoz-Fernandez, Marjorie Pion

**Affiliations:** 1 Laboratorio Inmuno-Biología Molecular, Hospital General Universitario Gregorio Marañón, Madrid, Spain; 2 Red Temática de Investigación Cooperativa Sanitaria del Instituto de Salud Carlos III (RETIC), Red de Investigación Sanitaria (RIS) HIV-Vaccine group, Madrid, Spain; New York University, United States of America

## Abstract

**Background:**

Human immunodeficiency virus type 1 (HIV-1) induces a general dysregulation of immune system. Dysregulation of B cell compartment is generally thought to be induced by HIV-related immune activation and lymphopenia. However, a direct influence of HIV-1 particles on B cells was recently proposed as the third pathway of B cells dysregulation.

**Methods/Principal Findings:**

We evaluated the direct and specific consequences of HIV-1 contact on activation, survival, proliferation and phenotype of primary B cells *in vitro*. Moreover, we examined expression of activation-induced cytidine deaminase (AID) mRNA that is responsible for class switch recombination (CSR) and somatic hypermutation (SHM). Here, we report that changes observed in cellular proliferation, phenotypes and activation of B cells could be caused by direct contact between HIV-1 particles and primary B cells *in vitro*. Finally, direct HIV-1-derived B cells activation led to the increase of AID mRNA expression and its subsequent CSR function was detected *in vitro*.

**Conclusion/Significance:**

We showed that HIV-1 could directly induce primary B cells dysregulation triggering phenotypical and functional abilities of B cells *in vitro* that could explain in some extent early B-cell abnormalities in HIV disease.

## Introduction

B cells are a critical component of the adaptive immune system, by producing highly specific antibodies and by establishing a CD27+ memory B cells. They are providing a specific and a long-term protection from an extensive range of pathogens [1], [2]. During HIV infection and in humoral immunity context, initial observations revealed that patients with acquired immune deficiency syndrome (AIDS) could exhibit hyperimmunoglobulinemia, increasing expression of cell-activation markers, depletion of memory B cells inducing ineffective recall responses, polyclonal B-cell hyperactivity, and altered differentiation of naïve B cells that could result in impaired immunoglobulin class switch recombination (CSR), and thus production of nonspecific immunoglobulin (Ig)G, IgE and IgA antibodies [3], [4], [5], [6], [7], [8]. All of these processes finally provoke defective responses to opportunistic pathogens and vaccines [7], [9]. The final step of this general B cells dysregulation could be the exhaustion of B cells compartment [10]. In general, HIV-1-related humoral defects are thought to originate from general immune activation and from the progressive CD4+ T cells lymphopenia since optimal and specific B cell activation needs B and T cells contact [11], [12], [13]. Until now, restoration of CD4+ T cells by antiretroviral therapy has not fully re-established antigen-specific IgG, IgE and IgA responses and memory B cells [14], [15], [16], [17], which suggests an additional B cells dysregulation pathway. Consistent with that possibility, few articles have shown that some viral proteins could be directly implicated in B cells dysfunction through gp120:DC-SIGN interactions, HIV-1 Nef delivered to B cells via infected macrophages, through mannose C-type lectin receptors or through CD21 [18], [19], [20], [21], [22], [23]. Moreover, complement receptor CD21 could not only bind HIV-1 *in vitro* and *in vivo* but could as well facilitate CD4+ T cells infection since B cells-associated HIV-1 is far more infectious for T cells than is free virus [22], [24], [25]. In consequence, and without evidence of direct infection, B cells could directly be dysregulated by HIV-1, if cells transport HIV-1 particles attached at their surface. How HIV-1 permits to dysregulate indirectly or directly B-cell *in vivo,* it is not still well defined albeit incorporation of CD40L into virion membrane during HIV budding could be one the B-cell activation process [26], [27], [28]. In fact, it is not completely clear if only CD40L-associated with HIV-1 or other human or viral compounds could be involved in general B cells dysregulation and thus helping the HIV-1 spreading in patients.

In this study, we first confirmed the rapid phenotype dysregulation and activation of peripheral primary B cells after direct HIV-1 contact. We then examined the survival rate of HIV-associated B cells when cultured *in vitro*. Interestingly, human primary B cells survival rate and cellular proliferation were increased when B cells were put in contact with HIV-1. Moreover, expression level of AID mRNA in human primary B cells was highly increased and its subsequent IgM/IgE; IgM/IgA and IgM/IgG class switch was detected *in vitro*. These results may highlight a possible relation between HIV-1 infection and B cells hyperactivation, loss of memory B cells or hyperglobulinemia.

## Results

### B Cells Survival in vitro

**Figure 1:**
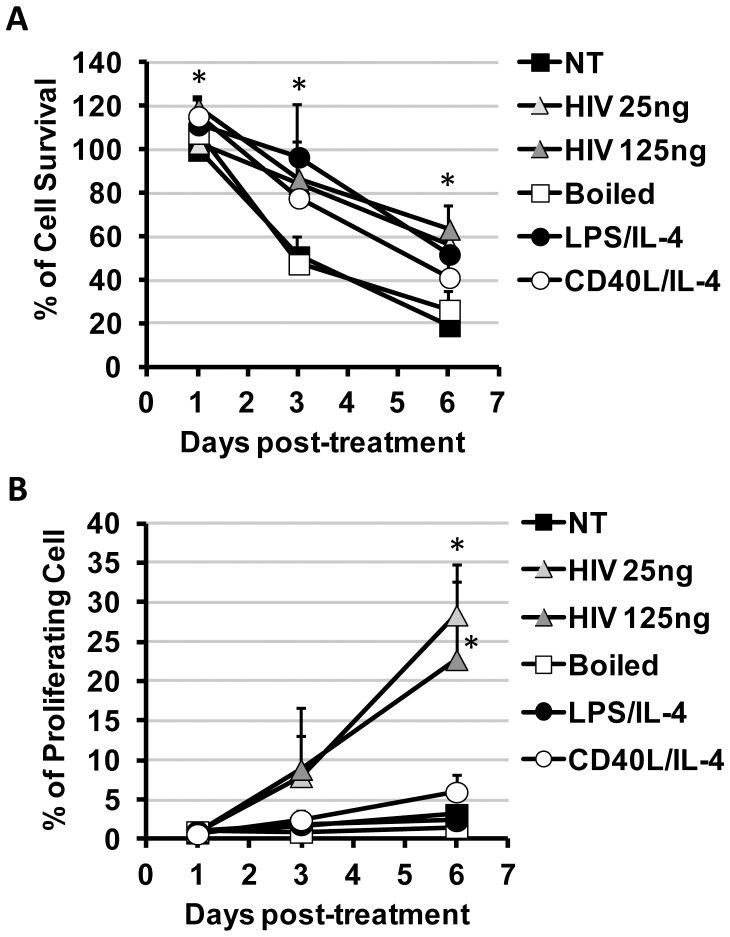
B cells survival and proliferation. B cells survival (A) and proliferation (B) were followed in *vitro*. (A) B cells were non-treated (NT) or treated with HIV_NL4-3_ at 25 ng or 125 ng of p24^gag^, with 125 ng of p24^gag^ boiled HIV_NL4-3_, with CD40L/IL-4or with LPS/IL-4. After 1, 4 and 6 days of culture, cells were collected and labeled with 7AAD, % of living cells were quantified in 7AAD negative population and % of cell survival was calculated as  =  (treated living cells / NT living cell at day 1 *100). (+SD; * = p<0.05 for both HIV-treatment conditions, LPS/IL-4 and CD40L/IL-4 conditions in comparison to NT condition)(B) After 1, 3 or 6 days post-treatment, percentage of proliferation was detected in CFSE-labeled B cells as the percent of B cells that lost their CFSE staining (See Figure S2). (+SD; * = p<0.05 for both HIV-treatment conditions in comparison to NT condition). Mean of 7 experiments (125ng of p24^gag^, 125ng of p24^gag^ boiled HIV_NL4-3_ or LPS/IL-4 condition), of 5 experiments (CD40L/IL-4 condition) or 12 experiments (NT or 25 ng of p24^gag^ conditions) shown.

After B cells isolation from PBMC of healthy volunteers, B cells were cultured *in vitro* in culture medium with or without stimulant factors. Percentages of living cells were followed by 7-AAD labeling by flow cytometry [29]. As expected the half-life of non-treated B cells culture was rather short. Surprisingly, after 6 days of treatment B cells survival was better when cells were treated with HIV-1, with CD40L/IL-4 or with LPS/IL-4 where an average of 40–60% of living B cells in comparison with boiled HIV-1 condition (corresponding to denatured HIV-1-derived antigens) or with non-treated cells (Figure 1A) as NT condition at day 1 corresponded to 100% of B-cell survival. We produced HIV-1 into cells MT2 that did not contain CD40L, confirmed by flow cytometry (Figure S1). Therefore, the HIV-1 produced was free of CD40L at their surface. Thus, a better B cells survival related to HIV-1 was not due to the presence of CD40L at the surface of virions.

To know if this survival rate was due to a resistance to apoptosis or to proliferation, we had submitted B cells to a CFSE pre-treatment before cultivating them. Proliferation was followed by the loss of CFSE signal by flow cytometry (Figure S2). Proliferation was detected at day 6 when B cells were treated with HIV_NL4-3_ but not when treated with LPS/IL-4 (Figure 1B). It was interesting to note that CD40L/IL-4 induced a slight proliferation even non significant of B-cell in comparison to NT condition (6.06±1.90% and 3.25±1.00% respectively) that could be considered as residual loss of CFSE. Summarizing, although LPS/IL-4 and CD40L/IL-4 treatments caused a better B cells survival in comparison with non-treated cells, B cells did not show a proliferation profile suggesting that survival signal pathways initiated by HIV_NL4-3_ or LPS/IL-4 could be independent.

### B Cells Activation

**Figure 2:**
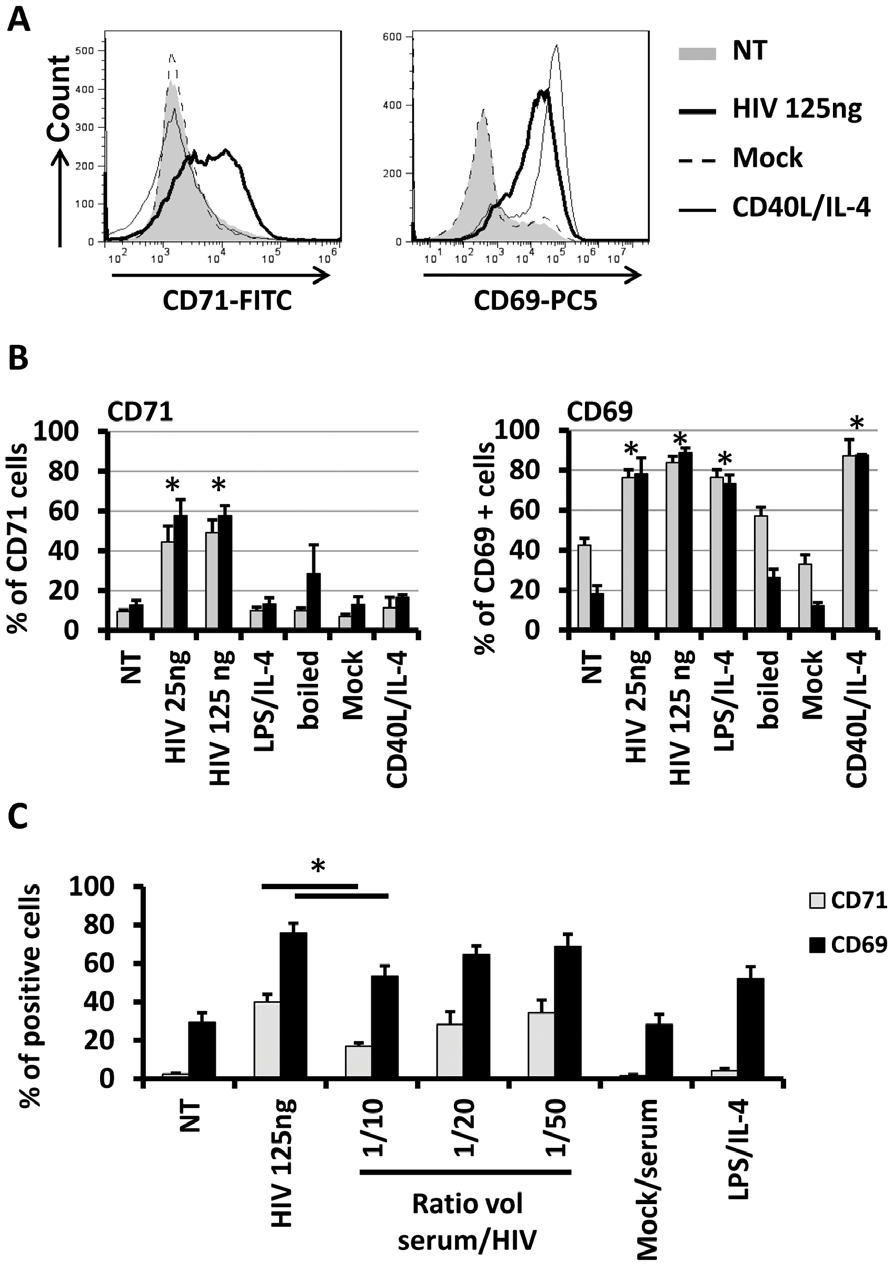
Expression of activation markers on B cells. (A) Histogram plots of CD71 (left panel) and CD69 (right panel) expression markers at the surface of B-cell. (B) B cells were NT or treated with 25 ng and 125 ng of p24^gag^ of HIV_NL4-3_, with 125 ng of p24^gag^ of boiled-HIV_NL4-3_, with LPS/IL-4, mock-treated or treated with CD40L/IL-4. At day 1 (grey bars) or day 4 (black bars) post-treatment, CD71 and CD69 surface markers were followed by flow cytometry. Mean of 7 individuals donors excepted for mock and CD40L/IL-4 conditions (3 individuals donors) (+SD; * = p<0.05 in comparison to NT for day 1 or 4 post-treatment). Mock corresponded to the supernatant of MT2 non-infected cells. (C) B cells were NT or treated with 125 ng of p24^gag^ of HIV_NL4-3_, with 125 ng of p24^gag^ of HIV_NL4-3_ treated with anti-HIV serum (Vol/Vol), mock-treated/anti-HIV serum or treated with LPS/IL-4. At day 1 post-treatment percentage of CD71 and CD69 were followed by flow cytometry. Mean of 3 individuals donors is represented (± SD; * = p<0.05).

We researched whether the increased survival ability was associated with the cellular activation. We compared the expression of B cells surface activation markers CD69 and CD71 by flow cytometry. B cells treated with HIV_NL4-3_ exhibited higher expression of CD69 and CD71 very early after activation at 24 h post-culture (Figure 2A) (83.82±2.90% and 49.16±6.38% of positive cells for 125 ng of p24^gag^, Table S1, Figure 2B) that was significantly different in comparison with non-treated B cells (42.44±3.50% of positive cells for CD69 and 9.57±0.84% positive cells for CD71, Figure 2B; Table S1). After 4 days of treatment, expression level of CD69 and CD71 were still significantly higher when B cells were treated with HIV_NL4-3_ when compared with non-treated or in mock treated cells (Figure 2B; Table 1).

**Table 1:** B-cell subpopulations after 4d of HIV_NL4-3_ treatment.

Percentage of B-cell populations after 4 days of contact with HIV_NL4-3_						
B-cell populations	NT	125 ng-HIV	Mock SN	boiled-HIV	LPS/IL-4	CD40L/IL-4
*Multiple labelling*						
Immature	**2.68^d^** (±0.67)	16.06*^a^ (±3.58)	**2.23^a^** (±0.40)	**3.84^a^** (±1.35)	**2.66^a^** (±1.03)	**2.27^a^** (±0.79)
Naive mature	**16.53^d^** (±2.95)	42.08*^a^ (±5.85)	**31.29^a^** (±5.91)	**20.87^a^** (±4.29)	**10.11^a^** (±3.75)	**13.83^a^** (±2.05)
Activated mature	**13.28^d^** (±0.60)	1.59^*a^ (±0.65)	**9.05^a^** (±1.73)	**7.93^a^** (±1.14)	**18.01^a^** (±2.36)	**17.35^a^** (±1.24)
Resting memory	**19.89^d^** (±2.48)	4.32^*a^ (±1.06)	**22.14^a^** (±4.76)	**25.28^a^** (±5.31)	**19.28^a^** (±4.51)	**19.32^a^** (±3.24)
Exhausted tissue like memory	**28.98^d^** (±3.12)	13.46^*a^ (±3.04)	**24.06^a^** (±3.59)	**19.99^a^** (±3.97)	**23.96^a^** (±3.01)	**30.18^a^** (±1.82)
Plasmablast	**0.65^d^** (±0.21)	**0.33^a^** (±0.14)	**1.35^a^** (±0.95)	**0.36^a^** (±0.14)	**0.80^a^** (±0.22)	**1.11^a^** (±0.33)
*Single labeling*						
CD27+	**39.91^f^** (±3.81)	9.85*^f^ (±3.24)	**35.64^a^** (±5.46)	**39.17^b^** (±6.83)	**32.83^d^** (±6.42)	**44.57^a^** (±6.95)
CD21hi	**46.43^e^** (±4.31)	73.58*^e^ (±5.99)	**61.38^a^** (±7.00)	**61.21^a^** (±8.46)	**37.28^b^** (±6.57)	**40.58^a^** (±4.11)
CD10+	**16.22^e^** (±2.28)	**22.90^e^** (±4.17)	**8.58^a^** (±2.13)	**15.82^a^** (±3.35)	**19.21^b^** (±4.27)	**14.60^a^** (±1.93)
CD71+	**7.44^f^** (±1.67)	56.12*^f^ (±3.89)	**6.63^a^** (±1.69)	**12.48^a^** (±4.58)	**8.44^c^** (±2.09)	**10.54^a^** (±1.66)
CD69+	**27.93^f^** (±4.31)	93.65*^f^ (±2.00)	**18.44^a^** (±1.91)	**26.27^a^** (±5.85)	80.71*^c^ (±6.98)	90.35*^a^ (±4.85)
CD24hi	**22.41^a^** (±1.16)	8.96*^a^ (±1.31)	**21.05^a^** (±3.06)	ND	ND	**18.44^a^** (±3.25)
CD38hi	**5.56^a^** (±0.56)	**19.19^a^** (±9.18)	25.19*^a^ (±3.88)	ND	ND	**9.38^a^** (±2.53)

Expression of cell surface markers and B-cell subpopulations size on B cells at 4 days post-treatment. Average percentage of cell surface markers and B-cell subpopulations size on NT B cells or treated with 125 ng of p24^gag^ of HIV_NL4-3_, Mock, boiled-HIV_NL4-3_, LPS/IL-4 and CD40L/IL-4 after 4 days of treatment. Results obtained from at least 4(^a^), 6 (^b^), 7 (^c^), 8 (^d^), 10 (^e^), and 12 (^f^) individual donors (±SEM). NT for non-treated B cells, ND for Non-Determined (*; p<0.05 in comparison to NT).

To confirm that B cells activation was due to direct HIV particles contact with B cells, we treated virus stock with different concentration of anti-HIV neutralizing serum for 1 h before B cells treatment. HIV_NL4-3_ treatment with anti-HIV serum permitted to partially reverse B cells activation induced by non-treated HIV_NL4-3_ particles (Figure 2C). With the higher anti-HIV concentration serum, we reached around 30% of significant reversion for CD69 marker expression and more than 58% of reversion for CD71 marker expression (Figure 2C). Summing up, our data indicate that HIV_NL4-3_ have the ability to induce cellular activation detected by the increase of markers CD69 and CD71 expression at the surface of primary B cells *in vitro*. Moreover, this activation is restricted to HIV_NL4-3_ treatment for CD71 activation marker and restricted to HIV_NL4-3_, LPS/IL-4 and CD40L/IL-4 for CD69 marker suggesting that LPS/IL-4, CD40L/IL-4 or HIV-derived cellular activation could act through independent pathways.

### B Cells Phenotype Dysregulation in vitro

We determined whether the HIV_NL4-3_ or other stimuli could influence phenotype apart from activation markers at the surface of the B cells. We compared the expression of B cells surface markers CD21hi, CD10, CD27 and CD20 (as described in Figure S3) or of differentiation markers CD24hi or CD38hi after treatments. B cells treated with 125 ng of p24^gag^ HIV_NL4-3_ showed significant changes in immature subpopulation at 24 h of treatment (Table S1). Significant changes were detected essentially after 4 days of culture. Immature and naïve mature B-cell subpopulations were increased after 4 days of HIV_NL4-3_ treatment, while activated mature, resting memory and exhausted tissue like memory subpopulations showed a significant decrease in comparison with non-treated B cells (Table 1).

Because phenotype reversion from more differentiated B cells such as exhausted tissue like memory B cells through less differentiated B cells such as immature and naïve mature cells were surprising, we checked the percentage of each markers expression at the surface of B cells. At 4 days post-treatment, expression of CD21hi was significantly higher when cells were treated with 125 ng of p24^gag^ of HIV_NL4-3_ in comparison with non-treated cells or mock condition (73.58±5.99%, 46.43±4.31% and 61.38±7.00%, respectively). Moreover, CD27+ populations were decreased after 4 days of treatment (9.85±3.24% for the condition 125 ng of p24^gag^ of HIV_NL4-3_, 39.91±3.81% for NT cells and 35.64±5.46% for the mock condition; Table 1). Finally, after 4 days of treatment, expression of the differentiation marker CD24hi was significantly lower when B cells were treated with 125 ng of p24^gag^ of HIV_NL4-3_ in comparison to non-treated or to the mock conditions (8.96±1.31%, 22.41±1.16% and 21.05±3.06% of positive cells, respectively; Table 1).

Summing up, we observed that the size of each B-cell subpopulations was modified when cells were subjected to HIV_NL4-3_ after 24 h or more deeply after 4 days of treatment. These B-cell subpopulation categories were directed from a major presence of less differentiated B cells subpopulations and minor end-differentiated B-cell populations. These unexpected results seemed to be essentially due to the increased presence of CD21hi and CD10 and loss of CD27 markers when B cells were treated with HIV_NL4-3_.

### AID Expression Dysregulation in in vitro B Cells Culture

**Figure 3:**
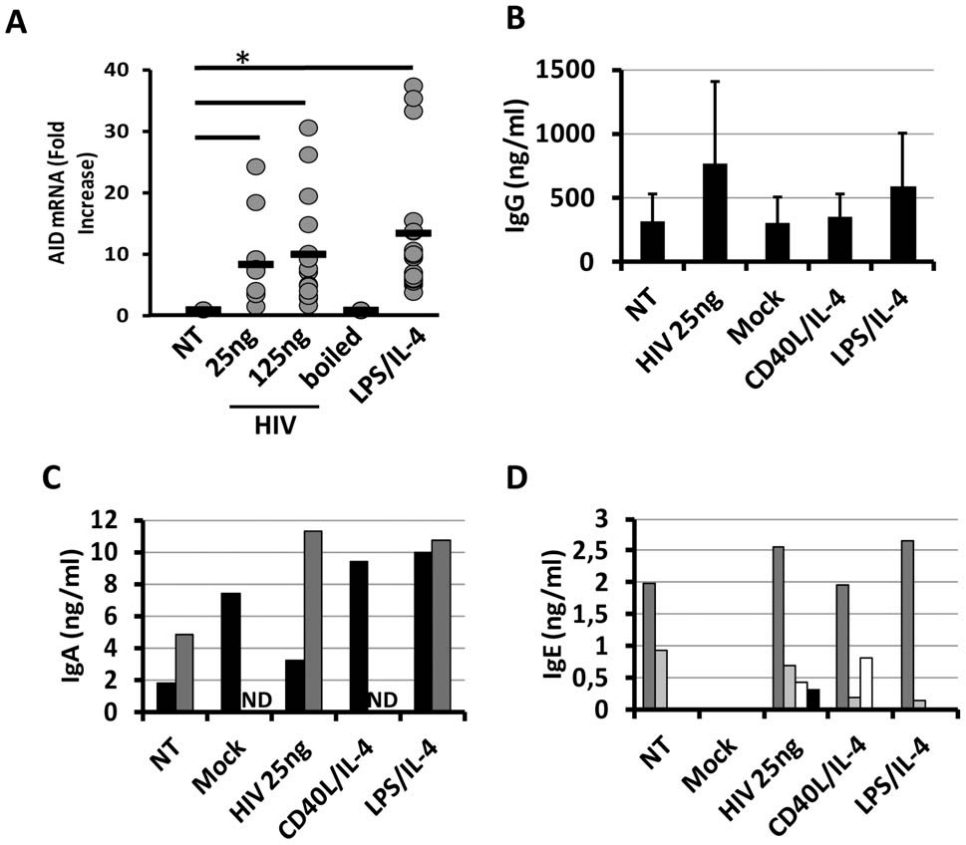
AID mRNA expression in B cells and Igs production. (A)AID mRNA expression was quantified at 24 h post treatment in B cells. NT or treated B cells were cultured *in vitro* and mRNA was extracted and AID mRNA expression was quantified by real-time PCR. Fold increase was calculated as ratio of AID mRNA expression in comparison to NT condition. Each grey dot represents one experiment. Black bar represent average fold increase. (* = p<0.05). IgG (B), IgA (C) and IgE (D) production were quantified in cell culture supernatant by ELISA kit. (B) Mean of 10 experiments was represented (+SD). (C, D) Only experiments with detectable level of IgA or IgE was shown where each bar represent the result obtained for one individual donor. (ND: not determined).

Given that activation of B cells is a normal process during pathogens infection and that activation precedes CSR and SHM processes induced by AID over-expression, we determined AID mRNA expression in B cells cultured *in vitro* by real-time PCR. mRNA from primary B cells cultured *in vitro* were extracted at 24 h after B cells treatment and only mRNAs showing a poor degradation profile were subjected to real-time PCR and AID mRNA expression levels were quantified. As shown in Figure 3A, HIV_NL4-3_ induced AID mRNA up-regulation after 24 h of treatment, B cells expressed an average of 10 times fold more AID mRNA than non-treated B cells (Figure 3A). Lack of mRNA AID induction in B cells when cells were treated with boiled-HIV_NL4-3_ showed that a full integrity of the virus particles was necessary to induce AID mRNA expression. Summing up, B cells showed an increased expression of AID mRNA that was comparable to LPS/IL-4 treatment very quickly after treatment with HIV_NL4-3_ particles.

### AID and CSR in in vitro B Cells Culture

**Figure 4:**
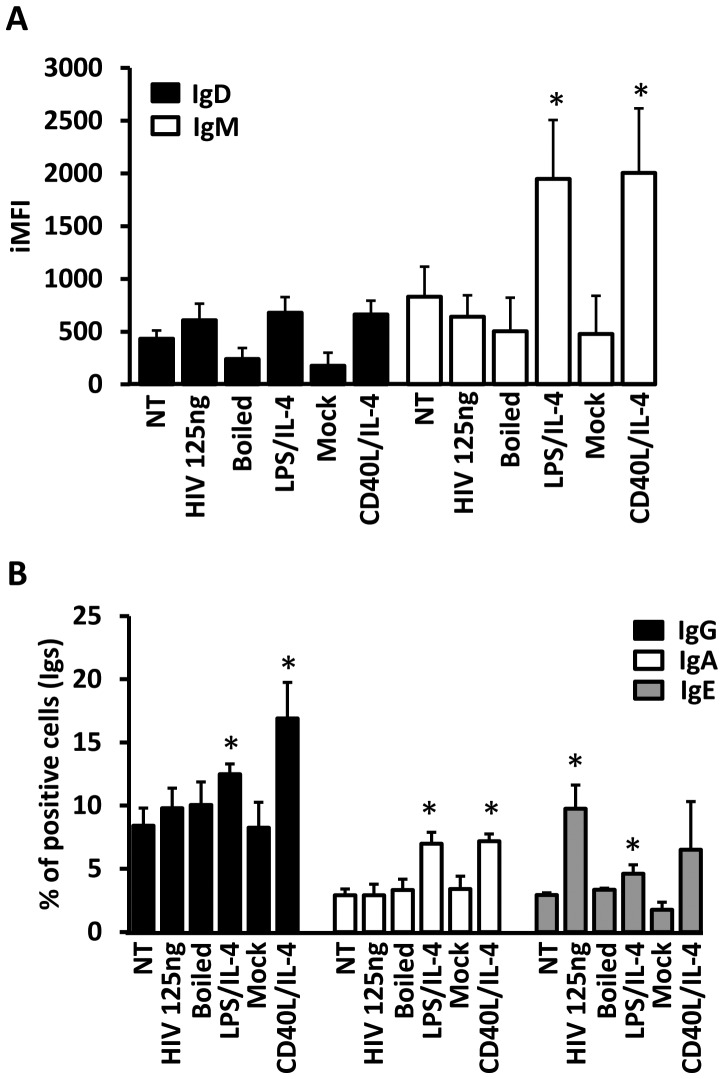
Class switch detected by intracellular labeling in in vitro B cells. (A) NT or treated B cells were cultured during 5 days. Intracellular CD19, IgM, IgD (A), IgG, IgA or IgE (B) markers were followed by flow cytometry. (A) IgD and IgM expression levels were followed in CD19+ population by percentage of positive cells for these markers *MFI of the same both markers ( = iMFI; integrated MFI). Mean of 5 individual donors (+SEM; * = p<0.05). (B) IgG, IgA and IgE expression levels were followed in CD19+ population by percentage of positive cells for each marker. Mean of 5 individual donors for IgG and IgA and 3 individual donors for IgE (+SEM; * = p<0.05).

We researched whether the increased AID mRNA expression level was associated with CSR and with the production of Igs in culture supernatant. Thus, we quantified IgG, IgA and IgE in the supernatant of the B cells culture after 5 days or treatment. IgG was detected on 10/10 of experiments and a slight non-significant increase was detected on supernatant of B cells culture when B cells were treated with 25 ng of p24^gag^ of HIV_NL4-3_ (Figure 3B). IgA was detected only in 2/10 experiments with a low level of IgA production (Figure 3C). On both positive experiments, only one showed increased level of IgA when cells were treated with HIV_NL4-3_ in comparison to non-treated condition. However, IgA level was increased in the same 2 experiments when cells were treated with LPS/IL-4. Finally, IgE was detected only in 4/10 experiments although with very low level of detection, less than 3 ng/ml (Figure 3D). Interestingly, on 4 experiments 2 showed undetectable level of IgE for non-treated condition, but exhibited positive level when treated with HIV_NL4-3_ (Figure 3D). Due to the low level of IgA and IgE detected in cell culture’s supernatant and because 5 days of incubation could be considered too short to induce detectable level of Igs in the supernatant of culture, we decided to label intracellularly treated B cells with CD19, IgM, IgD, IgA, IgE and IgG. Because great majority of changes for IgD and IgM expression levels were observed at the MFI level, we decided to quantify the integrated MFI that permitted us to analyze changes on percentage of positive cells for the studied marker and its MFI (iMFI = % of positive cells *MFI). Total IgM or IgD levels in CD19+ B cells treated with HIV_NL4-3_ were not significantly different in comparison with non-treated or mock conditions (Figure 4A). Only LPS/IL-4 and CD40L/IL-4 treatment induced significant changes for IgM expression level but not for the IgD expression level (Figure 4A). Figure S4 showed histogram plots of 2 representative donors for intracellular IgG, IgA and IgE markers. LPS/IL-4 treatment of B cells induced a significant change in CD19+/IgA, IgG and IgE expression levels in comparison to mock treated cells (Figure 4B). As previously described, CD40L/IL-4 treatment was inducing significant changes in CD19+/IgG and IgA expression level in comparison to non-treated or mock conditions (Figure 4B, Figure S4A-B) [30], [31]. Finally, B cells treatment with HIV_NL4-3_ induced only the significant increase of the IgE+ in CD19+ B cells (9.74±1.89% in comparison to non-treated condition that showed 2.91±0.18% of CD19+/IgE+, Figure S4C and Figure 4B). Summing up, HIV_NL4-3_ treatment seemed to be able to induce significant IgE expression whereas CD40L/IL-4 or LPS/IL-4 treatment induced significant increased production of IgG or IgA.

## Discussion

The objective of this study was to determine if HIV-1 particle by itself could affect key properties of human primary B cells. Summing up, we have found that these functional features of human primary B cells *in vitro* were adversely affected by the direct interaction with HIV_NL4-3_ particles.

Since several publications have shown that functional human surface markers CD40L could be found at the surface of the HIV-1, incorporated into HIV-1’s envelope membrane upon viral budding from infected cells [27], we determined presence of CD40L on virus producing cells and conclude that our HIV_NL4-3_ particles could not bear CD40L at their surface. Moreover, using as control HIV_NL4-3_ particles treated with a neutralizing serum anti-HIV-1 that blocked HIV-1 epitopes, we can assume that HIV-1 particles could directly activate B cells *in vitro* and be responsible for the increase of AID mRNA expression. As a consequence, our results of B cells dysregulation could not be explained by the presence of CD40L that is in contradiction with some articles that shown that B-cell activation is essentially due to the presence of CD40L at the surface of HIV-1 particles [26], [28]. Regarding our results we proposed that B cells could be activated, could express AID mRNA and could induce CSR in some extend independently of the CD40L/CD40 activation pathway. In B-cell/HIV-1 literature, some viral proteins were found to be bound to DC-SIGN, to CD21 or to mannose C-type lectin receptors thus our results showing importance of a native form of HIV-1 particles and its proteins could be explained by the necessity of viral particles to bound one or various cellular markers. However, we cannot rule out the possibility that other cellular factors present at the surface of HIV-1 particles could play a role in B-cell activation. Further experiments would be necessary to determine, which viral or cellular proteins are necessary for this strong B-cell activation described in this work.

We assume that results of activation, proliferation and differentiation found in this work could be associated to the non-antigenic stimulation of B cell regardless lymphocyte interaction as already determined *in vitro* [32], [33], [34]. In our study and in addition of rapid B cells activation and cellular proliferation, HIV-1 particles induced a dysregulation of B-cell phenotype such as lost of memory (CD27+) B cells. The loss of CD27 in B cells could explain previous results showing a loss of CD27+ memory B cells and a major presence of CD27- (naive) B cells in HIV-1-infected patients [14], [35], [36], [37]. Moreover, recent article showed that CD27 surface marker used to differentiate naïve from memory B cells should be challenged since CD27– B cells from seropositive patients could produced class switched and somatically hyper-mutated antibodies during chronic infection [38]. Such type of results was determined in other chronic infection such as hepatitis C virus (HCV) where CD27- B cells population was found increased in peripheral blood of persistently infected patient and where loss of memory B cells subset was due to a downregulation of CD27 expression and not to cell death [37]. Consequently, further experiments should be done in the aim to detect if the loss of some B cells subpopulations is due to the decrease of surface markers or due to selective B-cell subpopulations proliferation versus B-cell death.

Finally, in addition to rapid phenotypic alterations of B-cell subsets, increased expression of mRNA AID in B cells was indeed followed by subsequent CSR process through expression of IgG, IgE and IgA depending on the stimulation factor. It is important to note that IgE expression upon HIV-1 stimulation was observed when cells were labeled intracellularly and detected in B cells culture supernatants. Importance of these results could be related to the fact that unspecific IgE and IgG production were found in HIV-1 patient developing hyper-immunoglobulinemia E and G, autoimmune diseases and hypersensitivity observed in some HIV-1 patients [6], [8], [39]. Such patients are then unable to respond correctly to subsequent infections by facilitating the T cell depletion and by the production of autoreactive antibodies. Thus, our model could be essential to study mechanisms of the loss of specific humoral immunity in HIV+ patients independently of CD4+ T cells. Generally, B cells activation related to IgM, IgD or IgG production dictate the extent and efficacy of the antibody response, requiring a multitude of signals, including B-cell receptor activation, CD40 stimulation, and cytokines signals to initiate activation, differentiation, and proliferation. In our study, free of T cells and free of CD40L context, HIV-1 treatment of B-cell could induce cellular activation, proliferation and loss of principal markers of B cells differentiation. We postulate that events described in this work could participate at some extent to the general immune system dysfunction associated with HIV-1 infection. Moreover, the induction of non-antigenic CSR could suggest a limited ability for the host to induce a strong and specific B-cell response to both HIV-1 and other opportunistic pathogens. These findings should be taken in consideration during development of therapeutic HIV vaccines because of the large level of B-cell dysregulation.

## Materials and Methods

### B Cell Isolation from PBMC

Peripheral blood mononuclear cells (PBMC) were isolated on a Ficoll-Hypaque density gradient (Rafer, Zaragoza, Spain) from buffy coat obtained from transfusion centers of Albacete and Madrid following national guidelines following the current procedures of Spanish HIV BioBank [40]. B cells were purified using the CD19 MicroBeads (Miltenyi, Bergisch Gladbach, Germany), and contamination by other cell types was less than 3% (data not shown). Isolated B cells were cultured with RPMI 1640 medium (Biochrome, Berlin, Germany) supplemented with 5% heat-inactivated FCS, and antibiotics mix (125 µg/mL ampicilin, 125 µg/mL cloxaciclin and 40 µg/mL gentamicin; Sigma, St-Louis, MO, USA).

### Virus Stock Production

Virus stock HIV_NL4-3_ was produced by infection of MT2 cells (AIDS Research and Reference Reagent Program, Division of AIDS, NIAID, NIH: MT-2 from Dr. Douglas Richman [41]) with NL4-3 virus stock coming from previous transient transfection of pNL4-3 in 293T cells (ATCC, [42]). We produced parental X4 tropic HIV_NL4-3_ that encodes all known HIV-1 proteins [43] and physical titers were evaluated by quantification of HIV-1 p24*^gag^* by ELISA kit (Innogenetics, Gent, Belgium). Boiled virus was produced by heating HIV_NL4-3_ at 96°C for 10 min to obtain our negative control of activation as denaturated HIV-derived antigens.

### Culture and B Cells Treatment

B cells were treated with different amount of p24*^gag^* of infectious HIV_NL4-3_ (25 ng or 125 ng of p24*^gag^*/ 10^6^ B cells). Cells were as well treated with 20 µg/mL of Lipopolysaccharide (LPS) (Sigma-Aldrich) and 20 ng/mL of interleukine 4 (IL-4) (Immunotools, Friesoythe, Germany) or with 200 ng/ml of CD40L (eBioscience, San Diego, CA, US) and 10 ng/ml of IL-4 as positive controls of activation. Mock condition was defined by the use of non-infected MT2 cells supernatant. Blocking experiments were performed incubating 1/10, 1/20 and 1/50 (vol:vol) of HIV_NL4-3_ for 1 hour at 37°C before B cell treatment with anti-HIV neutralizing serum (AIDS Research and Reference Reagent Program: HIV-1 Neutralizing Serum (specify 1 or 2) from Dr. Luba Vujcic [44]). This serum was tested to neutralize several HIV-1 strains (see aidsreagent.org/reagentdetail.cfm?t = polyclonal_antibodies&id = 87). B cells survival was followed by flow cytometry using 0.5 µg/ml of 7-animo-actinomycin D (7AAD, Sigma-Aldrich) and B cells proliferation was followed by flow cytometry using 1 µM of carboxyfluorescein diacetate succinimidyl ester (CFSE) from CellTrace CFSE cell prolif kit (Invitrogen, Barcelona, Spain).

### Flow Cytometry for B Cells Purity and Phenotype

B cells were analyzed by flow cytometry analysis. Cells were stained for verification of B cells isolation purity along with activation and differentiation markers, with fluorescein (FITC)-labeled monoclonal Abs (MAbs): anti-CD21, anti-CD71, anti-IgD (Beckman Coulter) and anti-IgE (Thermo Scientific, Rockfold, IL, US). Phycoerythrin (PE)-labeled MAbs: anti-CD3, anti-CD10, anti-CD27, anti-CD38, anti-CD40L (Beckman Coulter) and anti-IgG (Miltenyi). R phycoerythrin-Texas-Red (named ECD)-labeled MAbs: anti-CD19 and streptavidin-ECD (Beckman Coulter). Biotin-conjugated anti-IgM (Beckman Coulter) this antibody was revealed by steptavidin-PC7 (Beckman Coulter). Phycoerythrin-Cyanin 5.1 (PC5)-labeled MAbs: anti-CD16, anti-CD27, anti-CD24 and anti-CD69 (Beckman Coulter) and anti-IgA (Jackson ImmunoResearch, Newmarket, UK). Phycoerythrin-Cyanin 7 (PC7)-labeled MAbs: anti-CD14 and anti-CD20 (Beckman Coulter). Intracellular anti-immunoglobulin labeling was performed with Citofix/Cytoperm kit (BD Biosciences, Franklin Lake, NJ, US) following manufacturer instructions. Cells were then fixed by adding 2% formaldehyde (Sigma-Aldrich) and analyzed by flow cytometry using a Gallios cytometer and data was analyzed using FlowJo 7.6.1 software (Ashland, OR, USA).

### RNA Isolation, AID mRNA Quantification by Real-time Polymerase Chain Reaction (Q-PCR)

RNA was extracted from 2×10^6^ cells with the Qiagen RNeasy Plus mini kit (Qiagen, Germantown, USA). RNA integrity was analyzed with Agilent 2100 bioanalyzer (Agilent Technologies, Waldbronn, Germany) using RNA Nano chips (Agilent Technologies). 100 ng of RNA was reverse transcribed to make cDNA (20 µl total volume) with the GoScript Reverse Transcription System (Promega, Madison, WI, USA). cDNA was used in the Q-PCR reaction with Brilliant II SYBR Green QPCR Master Mix (Agilent Technologies, Waldbronn, Germany). The Q-PCR reaction conditions were: 95°C 30 s, 60°C 1 min, 72°C 1 min for 40 cycles. The primers used to amplify mRNA of AID were: Forward Fw-5′-CGCGCCGGGGTGCAAATAGCCATC-3′, and Reverse Rv-5′-ACAGGGGCAAAAGGAT GCGCC-3′, and the primers to amplify mRNA of housekeepings were: Fw(YWHAZ)-5′-ACTTTTGGTACATTGTGGCTTCAA-3′, Rv(YWHAZ)-5′-CCGCCAGGACAAACCAGT AT-3′, Fw(β2M)-5′-TGCTGTCTCCATGTTTGATGTATCT-3′ and Rv(β2M)-5′-TCTCTGCTCCCCACCTCTAAGT-3′. AID mRNA levels detected by Q-PCR were normalized with YWHAZ and β2M, fold change was calculated following the 2^−ΔΔCt^ equation.

### Measurement of Igs in Cells Culture Supernatant

Total IgG, IgE and IgA in culture supernatants were measured using a human IgG, IgE and IgA enzyme-linked immunosorbent assay (ELISA) quantitation kit (Innovative Research, peary Court Novi, Mi, USA), according to the manufacturer’s specifications. Plates were read immediately at 450 nm with Synergy 4 reader (Biotek, Bad Friedrichshall, Germany).

### Statistical Analysis

The comparison between the surface marker levels and B cell subpopulations between non-treated B cells and treated B cells treated was realized using the non-parametric Mann-Whitney test, because the number of donors was low. All analyses were performed using SPSS 17.0 Inc. (IBM, Chicago, Illinois, USA).

## Supporting Information

Figure S1 **CD40L expression on MT2 cell line.** CD40L expression was quantified by flow cytometry in activated PBMC and MT2 cells. One of 2 representative experiments is shown. Numbers were the percentage of cells positive in each quadrant.
(TIF)

Figure S2 **B cell survival and proliferation gating.** CFSE-labeled B cells were treated for 6 days with 25 ng of p24^gag^ HIV, CD40L/IL-4 or NT. Cells were then labeled with 7AAD and fixed before flow cytometry analysis. Gate and number into the upper panels represent percentage of living cells (L). Cells gated in L were analyzed for CFSE presence (lower panel). Proliferating cells were cells that lost CFSE labeling. (Histogram dark grey: NT condition; light grey: HIV-treatment condition and very light grey: CD40L/IL-4 treated B cells).
(TIF)

Figure S3 **B cells subpopulations.** Subpopulations of B cells were determined by combination of CD21, CD10, CD27 and CD20 surface markers. CD21 low (gate A) and CD21 high (gate B) populations were fist determined. CD21high and CD21low were gated for CD10, CD27 and CD20. Thus, immature B cells (CD21hiCD10+CD27−, gate C), naïve mature (CD21hiCD10-CD27−, gate D), resting memory (CD21hiCD10-CD27+, gate E), activated mature (CD21loCD10-CD27+, gate F), long live plasma cells (CD21loCD10-CD27+CD20−, gate G) and exhausted tissue like memory (CD21loCD10-CD27-CD20+, gate H) subpopulations were detected and quantified by flow cytometry.
(TIF)

Figure S4 **Intracellular Igs labeling in B cells.** Two individual donors on 5 were represented for intracellular labeling of IgG (A), IgA (B) or IgE (C) after 5 days of treatment. Cells were first gated on CD19+ population.
(TIF)

Table S1 **Expression of cell surface markers and B-cell subpopulations size on B cells at 24 h post-treatment.** Average percentage of cell surface markers and B-cell subpopulations size on NT B cells or treated with 25 ng or 125 ng of p24^gag^ of HIV_NL4-3_, boiled-HIV_NL4-3_, and LPS/IL-4 after 24 h of treatment. Results obtained from at least 6(^a^), 7 (^b^) and 10 (^c^) individual donors (±SEM). NT for non-treated B cells (^*^; p<0.05 in comparison to NT).
(TIF)
