# Supplementary figures and images for: Direct Phenotypical and Functional Dysregulation of Primary Human B Cells by Human Immunodeficiency Virus (HIV) Type 1 In Vitro
Source: PLoS One. 2012 Jul 2;7(7):e39472. doi: 10.1371/journal.pone.0039472 (PMC3388069; doi:10.1371/journal.pone.0039472)

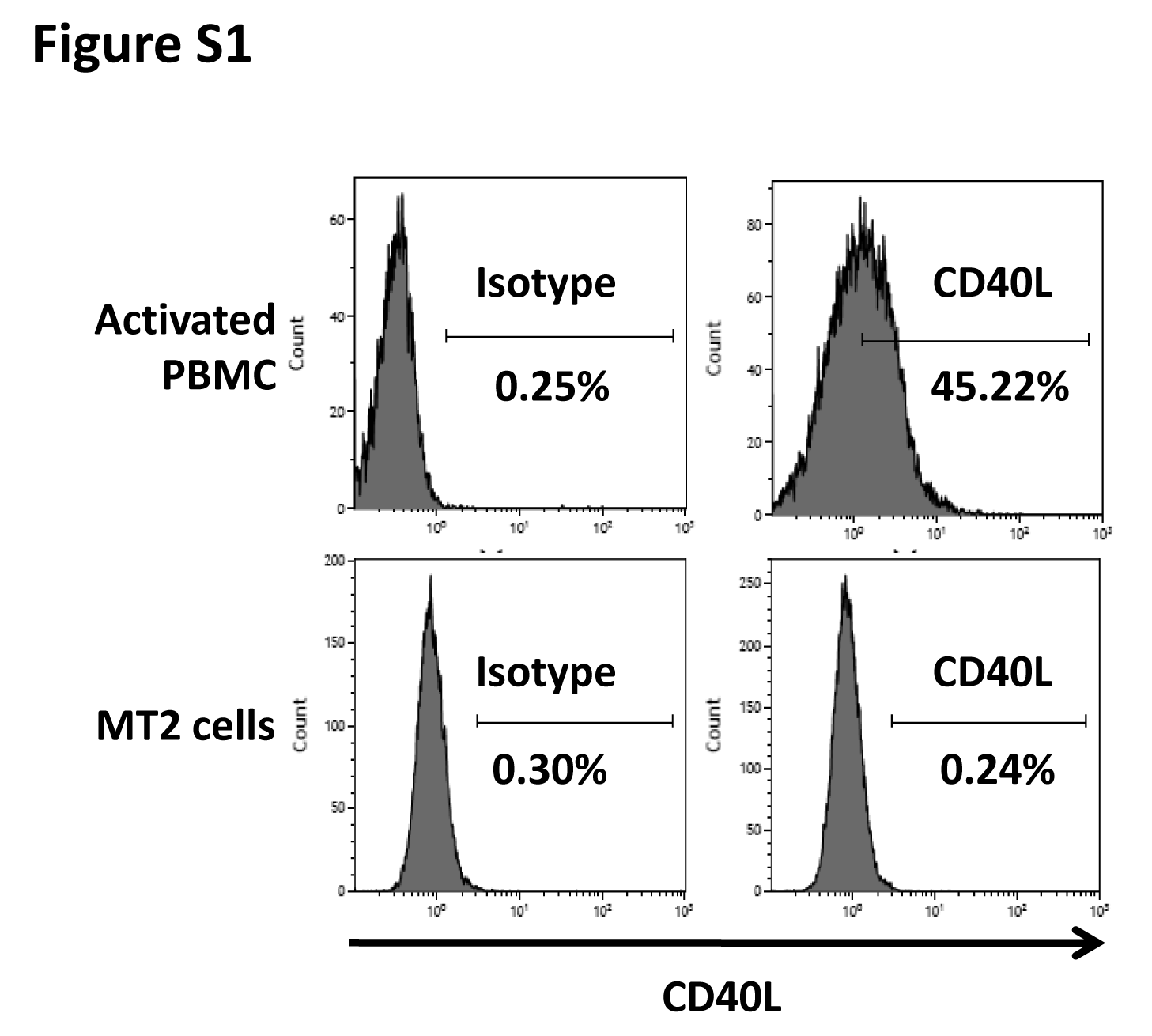

Supplement: Figure S1 — CD40L expression on MT2 cell line. CD40L expression was quantified by flow cytometry in activated PBMC and MT2 cells. One of 2 representative experiments is shown. Numbers were the percentage of cells positive in each quadrant. (TIF) [file pone.0039472.s001.tif]

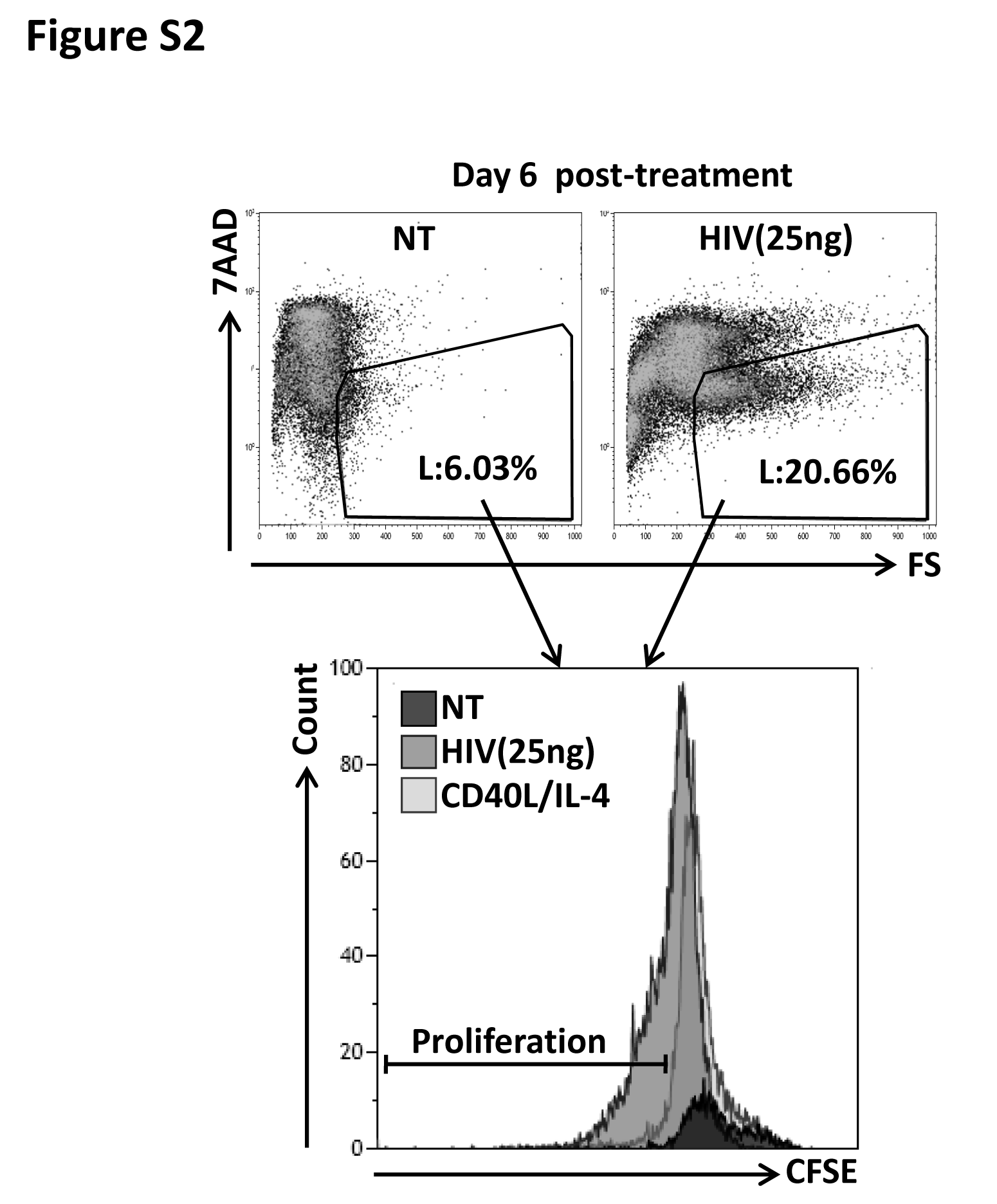

Supplement: Figure S2 — B cell survival and proliferation gating. CFSE-labeled B cells were treated for 6 days with 25 ng of p24gag HIV, CD40L/IL-4 or NT. Cells were then labeled with 7AAD and fixed before flow cytometry analysis. Gate and number into the upper panels represent percentage of living cells (L). Cells gated in L were analyzed for CFSE presence (lower panel). Proliferating cells were cells that lost CFSE labeling. (Histogram dark grey: NT condition; light grey: HIV-treatment condition and very light grey: CD40L/IL-4 treated B cells). (TIF) [file pone.0039472.s002.tif]

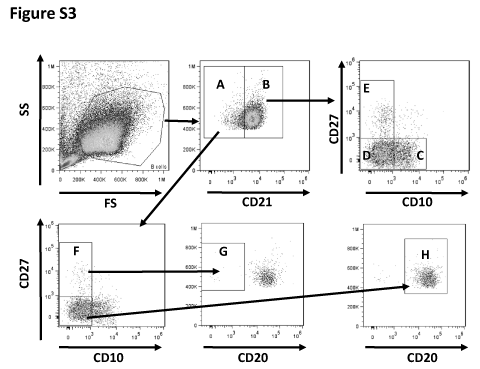

Supplement: Figure S3 — B cells subpopulations. Subpopulations of B cells were determined by combination of CD21, CD10, CD27 and CD20 surface markers. CD21 low (gate A) and CD21 high (gate B) populations were fist determined. CD21high and CD21low were gated for CD10, CD27 and CD20. Thus, immature B cells (CD21hiCD10+CD27−, gate C), naïve mature (CD21hiCD10-CD27−, gate D), resting memory (CD21hiCD10-CD27+, gate E), activated mature (CD21loCD10-CD27+, gate F), long live plasma cells (CD21loCD10-CD27+CD20−, gate G) and exhausted tissue like memory (CD21loCD10-CD27-CD20+, gate H) subpopulations were detected and quantified by flow cytometry. (TIF) [file pone.0039472.s003.tif]

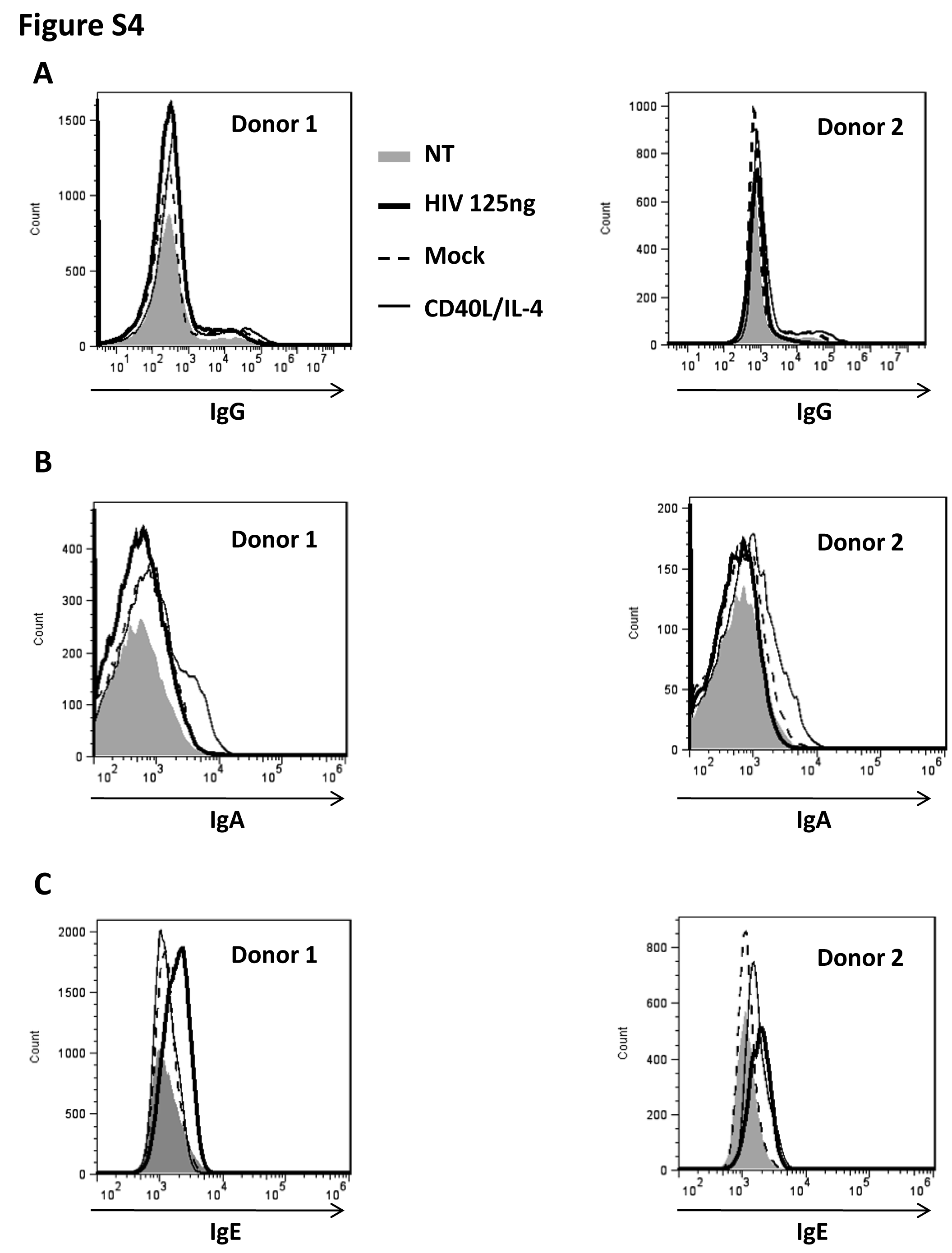

Supplement: Figure S4 — Intracellular Igs labeling in B cells. Two individual donors on 5 were represented for intracellular labeling of IgG (A), IgA (B) or IgE (C) after 5 days of treatment. Cells were first gated on CD19+ population. (TIF) [file pone.0039472.s004.tif]

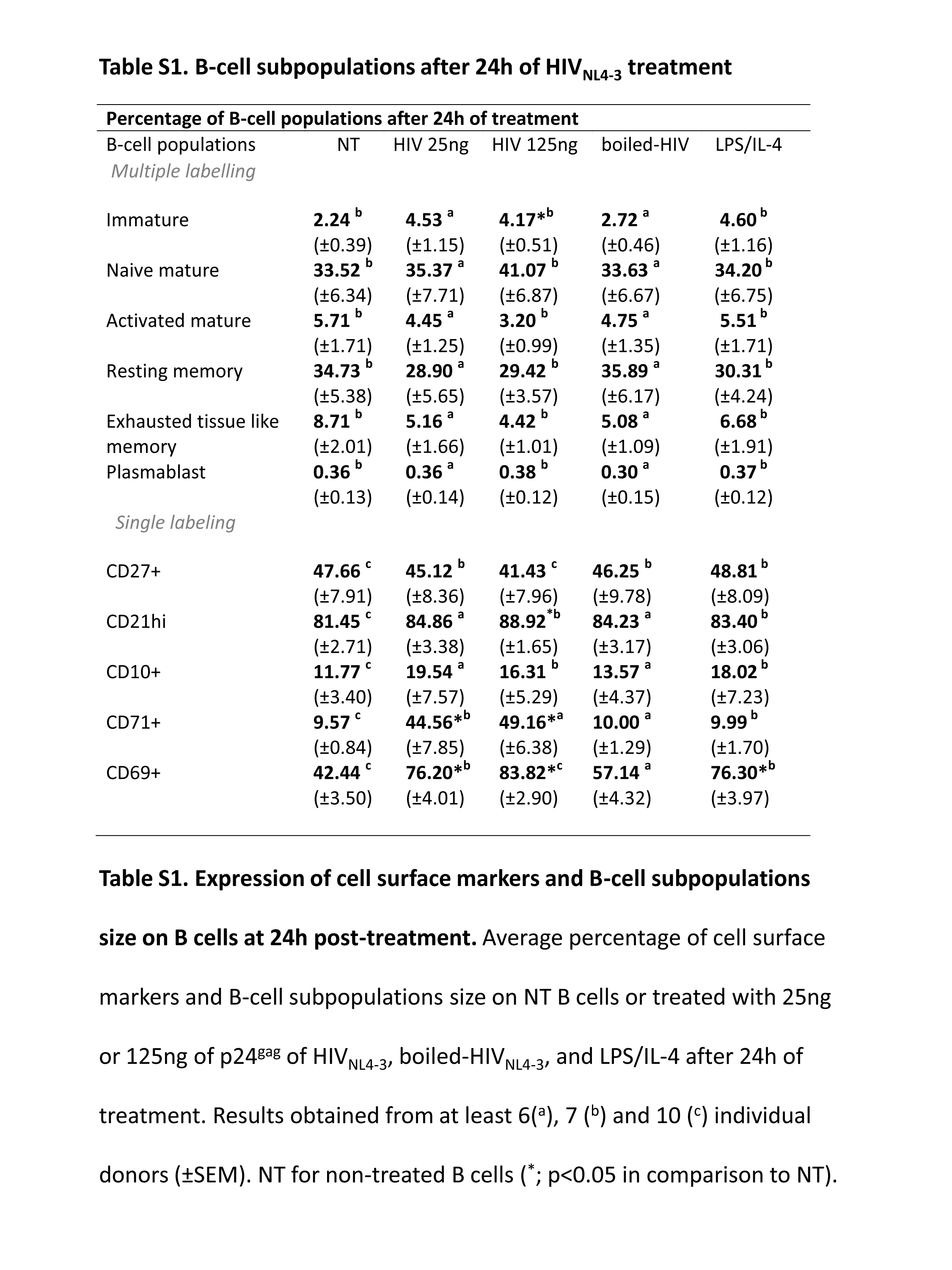

Supplement: Table S1 — Expression of cell surface markers and B-cell subpopulations size on B cells at 24 h post-treatment. Average percentage of cell surface markers and B-cell subpopulations size on NT B cells or treated with 25 ng or 125 ng of p24gag of HIVNL4-3, boiled-HIVNL4-3, and LPS/IL-4 after 24 h of treatment. Results obtained from at least 6(a), 7 (b) and 10 (c) individual donors (±SEM). NT for non-treated B cells (*; p<0.05 in comparison to NT). (TIF) [file pone.0039472.s005.tif]
